# Serological proteomic biomarkers to identify *Paracoccidioides* species and risk of relapse

**DOI:** 10.1371/journal.pone.0202804

**Published:** 2018-08-29

**Authors:** Tatiane Fernanda Sylvestre, Ricardo de Souza Cavalcante, Julhiany de Fátima da Silva, Anamaria Mello Miranda Paniago, Simone Schneider Weber, Bianca Alves Pauletti, Lídia Raquel de Carvalho, Lucilene Delazari dos Santos, Rinaldo Poncio Mendes

**Affiliations:** 1 Universidade Estadual Paulista (UNESP), Faculdade de Medicina de Botucatu, Botucatu, São Paulo, Brazil; 2 Universidade Federal de Mato Grosso do Sul (UFMS), Faculdade de Medicina, Campo Grande, Brazil; 3 Instituto de Ciências Exatas e Tecnologia (ICET), Universidade Federal do Amazonas (UFAM), Itacoatiara, Brazil; 4 Laboratório Nacional de Biociências, CNPEM, Campinas, São Paulo, Brazil; 5 Universidade Estadual Paulista (UNESP), Instituto de Biociência de Botucatu, Botucatu, São Paulo, Brazil; 6 Centro de Estudos de Venenos e Animais Peçonhentos (CEVAP), UNESP, Botucatu, São Paulo, Brazil; University of Minnesota, UNITED STATES

## Abstract

The sensitivity of the double agar gel immunodiffusion test is about 90% in patients with untreated paracoccidioidomycosis (PCM), but it is much lower in cases of relapse. In addition, serum from patients with PCM caused by *Paracoccidioides lutzii*, frequent in the Midwest region of Brazil, do not react with the classical antigen obtained from Pb B-339. These findings showed the need for alternative diagnostic methods, such as biological markers through proteomics. The aim of this study was to identify biomarkers for the safe identification of PCM relapse and specific proteins that could distinguish infections caused by *Paracoccidioides brasiliensis* from those produced by *Paracoccidioides lutzii*. Proteomic analysis was performed in serum from 9 patients with PCM caused by *P*. *brasiliensis*, with and without relapse, from 4 patients with PCM produced by *P*. *lutzii*, and from 3 healthy controls. The comparative evaluation of the 29 identified plasma proteins suggested that the presence of the immunoglobulin (Ig) alpha-2 chain C region and the absence of Ig heavy chain V-III TIL indicate infection by *P*. *lutzii*. In addition, the absence of complement factor B protein might be a predictor of relapse. The evaluation of these proteins in a higher number of patients should be carried out in order to validate these findings.

## Introduction

Paracoccidioidomycosis (PCM) is a systemic disease caused by thermodimorphic fungi of the *Paracoccidioides brasiliensis* complex–*P*. *brasiliensis* and *P*. *lutzii* [1]. PCM is confined to Latin America and is endemic in an area that extends from Mexico to Argentina [2], with a higher incidence in Brazil, where it is frequently diagnosed in the state of São Paulo [3]. The recent identification of PCM caused by *P*. *lutzii* explained the low frequency of positive serological tests in these patients when the Pb B-339 antigen, a *P*. *brasiliensis* fungus, was used. This finding demonstrated the difficulty of performing both the serological diagnosis of PCM by *P*. *lutzii* and the serological follow-up during its treatment [4–6].

Despite the effective treatment of PCM caused by *P*. *brasiliensis*, quiescent fungi can lead to relapse, usually about five years after discontinuation of the treatment, in both acute/subacute and chronic forms [7]. Evaluations performed at admission have not been able to identify cases that progress to relapse. In addition, the recent identification of *P*. *lutzii* has required studies to identify PCM caused by every species. That study revealed that the sensitivity of the double immunodiffusion agar gel reaction (DID) was only 45% at relapse, and that the enzyme-linked immunosorbent assay (ELISA) was slightly better (65%), but with a sensitivity level much lower than that observed in treatment-naive patients [7].

The studies that used a proteomic approach focused on the identification of *P*. *brasiliensis* proteins [8–10] and *P*. *lutzii* [11]; however, they did not evaluate serum proteins from PCM patients.

The aim of this study was to use a proteomic approach to identify proteins that could differentiate infections caused by *P*. *brasiliensis* from those produced by *P*. *lutzii*, as well as to identify a biomarker that could foresee patients who are prone to relapse.

## Subjects and methods

### Patients

A prospective paracoccidioidomycosis control surveillance has been performed since 1988 at the Clinical Mycology Outpatient Service of the Tropical Diseases Area of the Faculdade de Medicina de Botucatu–São Paulo State University (UNESP) and since 1980 at the Infectious and Parasitic Diseases Service of the Faculdade de Medicina—Federal University of Mato Grosso do Sul (UFMS). The data have been stored initially in specifically prepared file cards and then transferred to a computer system. Serum samples from these patients were appropriately stocked, frozen and stored in a freezer at –80°C.

A retrospective study was performed in 13 PCM-patients, characterized as either confirmed or probable cases regarding Mendes criteria [12], and 3 healthy controls from Botucatu region. Treatment compliance, avoidance and/or stopping alcohol intake and smoking were reiterated to all patients at all outpatient visits

### Inclusion criteria

#### *A*. PCM cases caused by *P*. *brasiliensis*

Confirmed cases were characterized by the presence of a suggestive clinical picture and the identification of typical *Paracoccidioides* sp. yeast forms in one or more clinical materials, with or without a positive double agar gel immunodiffusion test (DID) using an antigen prepared from Pb B-339 [13]. We also included probable cases, characterized by the presence of a suggestive clinical picture and antibodies detected by DID, using antigen prepared from Pb B-339, but without a mycological confirmation. These characterizations were made upon the patient’s admission to the Faculdade de Medicina de Botucatu (UNESP), as to Mendes et al. criteria [12].

#### B. PCM cases caused by *P*. *lutzii*

We included confirmed cases, which were characterized by the presence of a suggestive clinical picture, the identification of typical forms of the *Paracoccidioides* sp yeast phase in one or more clinical materials, the absence of the serum antibodies by DID using the Pb B-339 antigen, and the identification of serum antibodies by DID using cell-free antigen (CFA) obtained from EPM-208 strain of P. *lutzii*, performed at the Federal University of São Paulo, Zoilo Pires de Camargo Service [6].

This characterization was carried out at patient admission to the Infectious and Parasitic Diseases Service of the Faculdade de Medicina–Federal University of Mato Grosso do Sul (UFMS).

#### Exclusion criteria

The presence of other systemic diseases of infectious, inflammatory, or neoplastic origin as co-morbidities, pregnancy and lactation were considered exclusion criteria.

#### Classification of clinical forms

The clinical forms and severity of each patient were classified by the specialist in infectious diseases who attended these patients, regarding Mendes (1994) [14] and Mendes et al. (2017) [12].

#### Appropriate treatment and definition of relapse

The treatment was considered appropriate when the patient progressed until reaching the apparent cure. The apparent cure includes a clinical cure (the disappearance of the symptomatology characterizing the disease, and normalized erythrocyte sedimentation rate), as well as a serological cure (persistent negative DID for one year under consolidation antifungal treatment), radiological cure (the disappearance of compatible lung lesions upon activity, substituted by residual lesions, such as fibrosis and emphysema) and, finally, the maintenance of these conditions for two years after discontinuation of the antifungal compound [12].

Relapse was characterized by the resurgence of PCM-compatible signs and symptoms in initially confirmed or probable cases associated with the identification of typical forms of the yeast phase of *P*. *brasiliensis* in any clinical specimen and/or the identification of specific antibodies by DID. Then, these types of relapse were clinical and mycological, clinical and serological or clinical, mycological and serological, depending on the laboratory results. Patients presenting suggestive clinical picture, but with negative mycological and serological tests, were considered relapsed cases only when they reached apparent cure following cotrimoxazole (CMX) treatment. This relapse was considered of the clinical type. None of the relapsed cases presented any laboratory evidence of disease of another etiology.

#### Study groups and research design

Group 1 (G1): consisted of five patients from the Botucatu region, with PCM caused by *P*. *brasiliensis*; these individuals presented a late relapse;

Group 2 (G2): consisted of four patients from the Midwest region of Brazil, with PCM caused by *P*. *lutzii*;

Group 3 (G3): was comprised of four patients from the Botucatu region, with PCM caused by *P*. *brasiliensis*; these individuals did not relapse; and

Group 4 (G4): consisted of three healthy individuals living in the Botucatu region; they were blood donors from the Blood Center of Botucatu.

The evaluation of patients from G1, G2, and G3 was performed on admission, before the introduction of the antifungal treatment. Table 1 presents the characterization data of the patients regarding gender, age, clinical form, and diagnosis and of the healthy controls, as to gender and age.

**Table 1:** Characterization of study groups according to gender, age and clinical form.

Groups	Individual	Gender	Clinical form	Age (years)	Diagnosis	
				before treatment	before treatment	at relapse
**Group 1** **Patients with** ***P*. *brasiliensis* and relapse**	01	M	CF	42	MD / CP sputum	Clinical / MD
	02	M	CF	50	HP oral mucosa	Clinical–cure with CMX*
	03	M	CF	34	CP sputum	Clinical–cure with CMX
	04	F	AF	15	CP lymph node	Clinical / HP lymph node
	05	F	AF	85	CP linfonodo / HP skin	Clinical / HP skin
**Group 2** **Patients with** ***P*. *lutzii***	06	M	CF	53	MD oral mucosa	-
	07	M	CF	43	HP oral mucosa	-
	08	M	CF	38	HP oral mucosa	-
	09	M	CF	53	HP skin / MD sputum	-
**Group 3** **Patients with** ***P*. *brasiliensis*, without relapse**	10	M	CF	54	MD sputum / HP oral mucosa	-
	11	M	CF	55	HP oral mucosa	-
	12	M	AF	38	HP lymph node	-
	13	M	AF	17	MD / HP skin	-
**Group 4** **Healthy individuals**	14	M	-	35	-	-
	15	M	-	29	-	-
	16	F	-	42	-	-

CF- chronic clinical form; AF—acute/subacute clinical form; M- male; F- female; *P*. *brasiliensis*—*Paracoccidioidesbrasiliensis*; *P*. *lutzii*—*Paracoccidioideslutzii*; MD- mycological direct; CP sputum-cytopathologic sputum; HP oral mucosa—histopathology of oral mucosa; MDoral mucosa–Mycological direct of oralmucosa; CP lymph node-cytopathologic node; HP skin-histopathological skin; HP lymph node-histopathology of lymph node and CMX-cotrimoxazole

*patient presented a strongly positive ELISA test for the diagnosis of PCM relapse.

### Methods

#### Blood sampling and serum storage

Patients’ and individuals’ serum samples from the G1, G3, and G4 groups were drawn and stored in a freezer at –80°C at the Tropical Diseases Research Laboratory–Medical Mycology, Faculdade de Medicina de Botucatu–São Paulo State University (UNESP). The sera of G2 were assigned by Dr. Anamaria Mello Miranda Paniago, from the Infectious and Parasitic Diseases Service of the Faculdade de Medicina—Federal University of Mato Grosso do Sul (UFMS).

### Proteomic analysis

Proteomic analysis was performed in four steps: protein quantification, protein digestion in solution, peptide sequencing by mass spectrometry (MS), and data analysis to identify the proteins.

#### Protein auantification

The proteins present in the serum samples were quantitated in triplicate by Bradford’s method [15] (BioRad®; Protein Assay, cod. 500–0001), with bovine albumin (BSA) as the standard protein.

#### Protein digestion in solution

The samples were submitted to enzymatic digestion in solution. To this end, the reduction and alkylation steps were initiated using 10 mM dithiothreitol (DTT) and 45 mM iodoacetamide (IAA), respectively, both of which were solubilized in 50 mM ammonium bicarbonate solution. Then, the samples were submitted to hydrolysis in the presence of the enzyme trypsin (Promega Corporation, Madison, WI, USA) at a concentration of 1:50 (enzyme: substrate), solubilized in 50 mM ammonium bicarbonate buffer (pH 7.8), which occurred for 18 hours; it was interrupted with the addition of 1% (v/v) formic acid to the sample volume. The samples were then desalted using Sep-Pak Vac C18 (Waters Corporation, Milford, MA, USA) cartridges, reduced in SpeedVac^TM^ (ThermoFisher Scientific, Waltham, MA) and maintained at 4°C until the time of analysis by MS.

#### Peptide sequencing by MS

MS analysis was performed at the National Laboratory of Biosciences (LNBio), located at the National Center for Research in Energy and Materials (CNPEM) in Campinas, São Paulo State, Brazil. An aliquot (4.5 μL) of digested proteins was injected by an analytic column (C18 1.7 μm BEH 130) (100 μm× 100 mm) RP-UPLC (nanoAcquity UPLC; WatersCorporation), coupled with nano-electrospray tandem MS on a Q-Tof Premier API mass spectrometer (MicroMass; Waters Corporation) at a flow rate of 600 nL/minute. A trapping column (Symmetry C18; 180 μm× 20mm) was used for sample desalting at a flow rate of 5μL/minute over 2 minutes. The gradient was 2%–90% acetonitrile in 0.1% formic acid over 45 minutes. The instrument was operated in MS-positive mode, with data continuum acquisition occurring from m/z 100–2,000Da at a scan rate of 1 second, and an interscan delay of 0.1 seconds [16].

#### Data analysis for protein identification

The MS files were processed using the Mascot Distiller v.2.3.2.0 program (2009; Matrix Science Ltd., London, UK), along with the Mascot Server v.2.3.01.0 program (Matrix Science Ltd.).The following parameters were used: cleavage lost by trypsin; the fixed modification of carbamidomethylation; the variable modification of methionine oxidation; 0.1 Da mass tolerance for MS; and 0.1 Da mass tolerance for MSMS. Searches were conducted using the NCBI database (*Homo sapien* taxonomy, 33,695,097 sequences; available at http://www.ncbi.nlm.nih.gov/protein/?term=homo%20sapiens) containing 92,180 sequences and 36,693,332 residues. For protein quantification, the data were submitted to the Scaffold Q + analysis program (version 3.4.5; Proteome Software, Portland, OR, USA) obtaining normalized spectral count values for each identified protein.

After identification, the proteins were characterized according to the main functions they performed, and the primary proteins in each group were subsequently characterized. The networks of protein interactions against the differentially expressed proteins were analyzed using the STRING 10 tool.

### Statistical analysis

The results were presented as the mean and standard deviation. Student’s *t*-test was used to compare means for the dependent and independent samples.The null hypothesis was rejected when the error was equal to or less than 0.05.

For the quantitative evaluation, protein concentrations were defined as either reduced or differentially expressed following comparison with the control group.

### Research ethics committee

The study was approved by the Research Ethics Committee of the Faculdade de Medicina de Botucatu (UNESP), number 2.100.907, submission no. CAAE 21822813.9.0000.5411. Written informed consent for participation was given by the patient or parents.

## Results

### Identification of differentially expressed proteins in *P*. *brasiliensis* and *P*. *lutzii* infections before treatment

To identify the most abundant, differentially expressed proteins present in the serum of PCM patients caused by *P*. *brasiliensis* and *P*. *lutzii*, the samples were submitted to shotgun analysis, where all proteins were hydrolysed serially and subjected to the free-label analysis in a system liquid chromatography tandem MS (LC-MSMS). A total of 29 major proteins were detected and quantified. In the qualitative analysis, it was noted that the alpha-1-antichymotrypsin protein was not present in the control group (Table 2), but the immunoglobulin (Ig) alpha-2 chain C region was present only in the group of patients with PCM caused by *P*. *lutzii* (Table 2). The Ig heavy-chain V-III TIL was not present in patients with PCM caused by *P*. *lutzii* (Table 2), and CFB protein was not present in the group of patients with PCM caused by *P*. *brasiliensis* with relapse (Table 2). Finally, the alpha globin protein was not present in the group of patients with PCM caused by *P*. *brasiliensis* with relapse, nor was it present in the control group (Table 2).

**Table 2:** Qualitative analysis of serum proteins of patients with paracoccidioidomycosis, evaluated before treatment—G1, G2, G3 patients groups and healthy individuals—G4group.

Protein	Access code	[G1] *P*. *brasiliensis* with relapse		[G2] Patients with *P*. *lutzii*	[G3] *P*. *brasiliensis*, without relapse		[G4] Control group (n = 3)
		AF (n = 1)	CF (n = 2)	CF (n = 4)	AF (n = 2)	CF (n = 2)	
**1. *Serum albumin***	P02768.2	+	+	+	+	+	+
**2. *Transferrin***	P02787.3	+	+	+	+	+	+
**3. *Apoliprotein A-I***	P02647.1	+	+	+	+	+	+
***4*. *Haptoglobin***	P00738.1	+	+	+	+	+	+
**5. *Ig kappa chain C region***	P01834.2	+	+	+	+	+	+
**6. *Ig gamma-1 chain C region***	P01857.1	+	+	+	+	+	+
**7. *Ig lambda-2 chain C region***	P0CG05.1	+	+	+	+	+	+
**8. *Alpha-2-macroglobulin***	P01023.3	+	+	+	+	+	+
**9. *Ig alpha-1 chain C region***	P01876.2	+	+	+	+	+	+
**10. *Alpha-1-antitrypsin***	P01009.3	+	+	+	+	+	+
**11. *Hemopexin***	P02790.2	+	+	+	+	+	+
**12. *Ig gamma-2 chain C region***	P01859.2	+	+	+	+	+	+
**13. *Alpha-1-acid-glycoprotein***	P02763.1	+	+	+	+	+	+
**14. *Complement C3***	P01024.2	+	+	+	+	+	+
**15. *Apolipoprotein A-II***	P02652.1	+	+	+	+	+	+
**16. *Ig gamma-3 chain C region***	P01860.2	+	+	+	+	+	+
**17. *Ig gamma-4 chain C region***	P01861.1	+	+	+	+	+	+
***18*. *Vitamin D-BindingProtein***	P02774.1	+	+	+	+	+	+
**19. *Ceruloplasmin***	P00450.1	+	+	+	+	+	+
**20. *Complement C4-A***	P0C0L4.2	+	+	+	+	+	+
**21. *Alpha-1-antichymotrypsin***	P01011.2	+	+	+	+	+	-
**22. *Kininogen***	P01042.2	+	+	+	+	+	+
**23. *Ig alpha-2 chain C region***	P01877.3	-	-	+	-	-	-
**24. *Beta-globin***	P68871.2	+	+	+	+	+	+
**25. *Ig kappa chain V-III***	P04433.1	+	+	+	+	+	+
**26. *Beta-2-glycoprotein 1***	P02749.3	+	+	+	+	+	+
**27. *Ig heavy chain V-III TIL***	P01764.2	+	+	-	+	+	+
**28. *Complement factor B***	P00751.2	-	-	+	+	+	+
**29. *Alpha-globin***	P69905.2	-	-	+	+	+	-

+ present;—absent; AF—acute /subacute form; FC- chronic form and n- number of participants.

**Fig 1:**
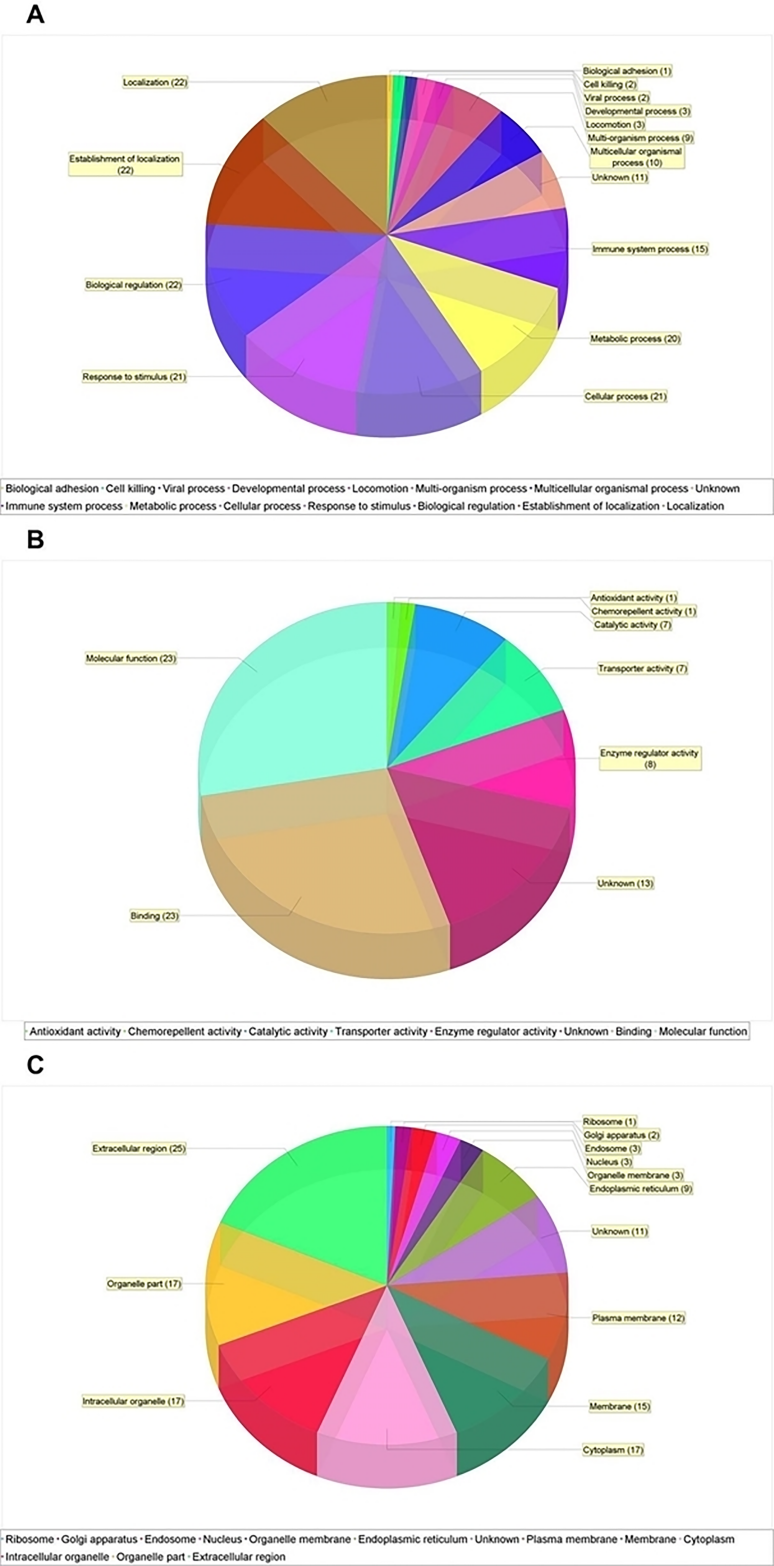
Classification of abundant proteins present in the human blood serum in infections of *P*. *brasiliensis* and *P*. *lutzii* before treatment according to their participation in a A) biological function, B) molecular function and C) cell components. The classification of proteins was analyzed by the STRING software according to the annotations deposited in the *Gene Ontology* database.

To obtain information about the functions that were evident in the cellular proteome, the identified proteins were attributed to different biological processes and molecular functions, and they were localized to specific cells based on the biological evidence from the NCBI protein database and the Gene Ontology database. The classification of these 29 abundant proteins can be visualized in Fig 1; among the main categories are transport proteins, immunomodulatory proteins, and proteins that act to activate/regulate the complement system, those that activate the coagulation/protease-inhibition pathway, those that transport/metabolize lipids, and those that inhibit proteases and the extracellular matrix.

**Fig 2:**
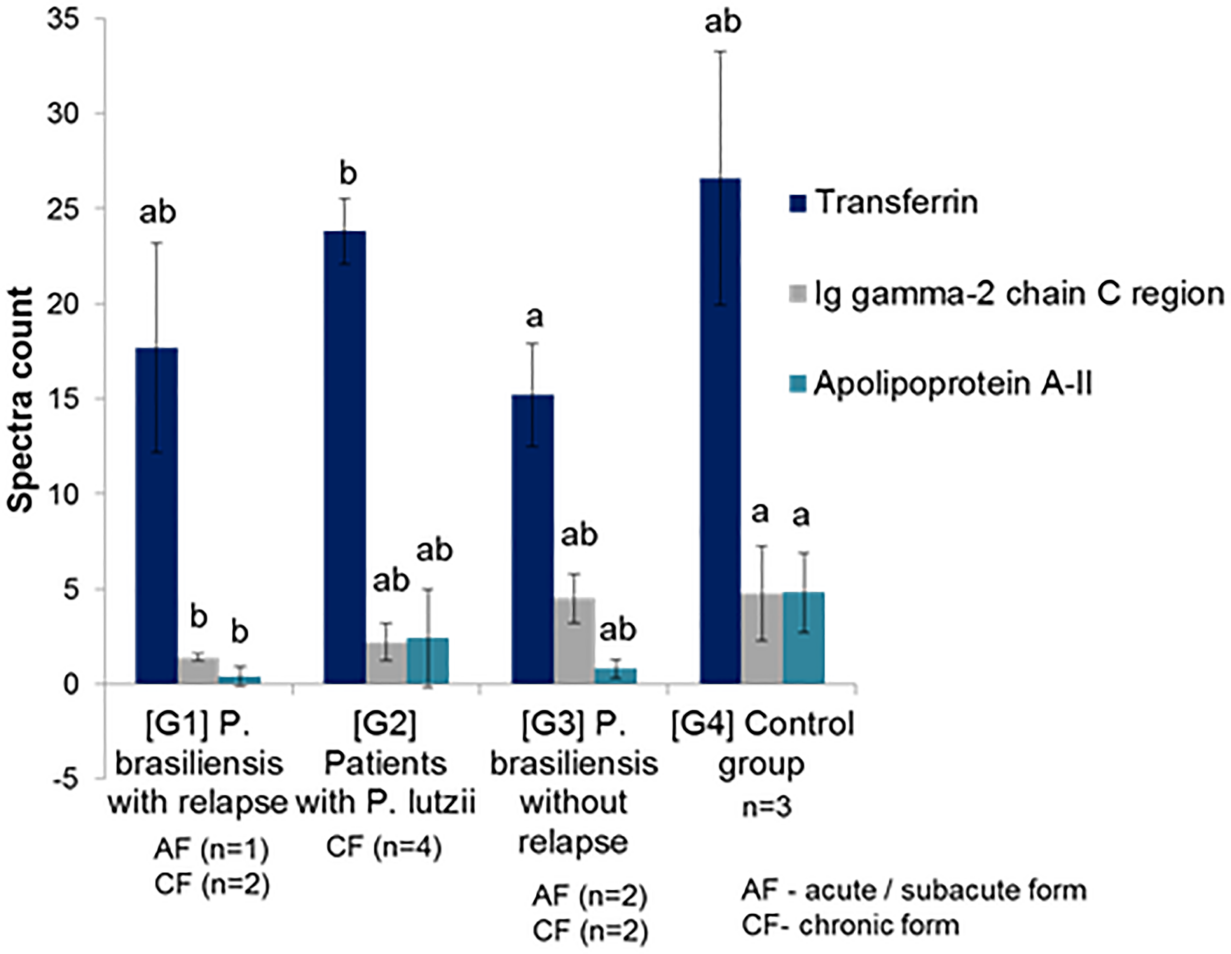
Comparison of serum proteins, presented as mean and standard deviation in patients with paracoccidioidomycosis, evaluated before treatment—G1, G2, G3 patients groups and healthy individuals—G4 group.

With respect to the quantitative analysis (Fig 2), statistically significant proteins with an adjusted *P*-value <0.05 (95% confidence) were considered differentially expressed among the sample groups (Table 3) more information can be found in S1 Table. The results of transferrin (Table 3) in patients with PCM caused by *P*. *brasiliensis* were evaluated in patients with AF and the CF as a whole because there was no difference regarding the clinical form, as in patients with as without relapse.

**Table 3:** Quantification of serum proteins by spectral counting, presented as mean and standard deviation in patients with paracoccidioidomycosis, evaluated before treatment—G1, G2, G3 patients groups and healthy individuals—G4 group.

Protein	Access code	Molecular mass (kDa)	Coverage rate (%)	[G1] *P*. *brasiliensis* with relapse AF (n = 1) / CF (n = 2)	[G2] Patients with *P*. *lutzii* CF (n = 4)	[G3] *P*. *brasiliensis* without relapse AF (n = 2) / CF (n = 2)	[G4] Control group (n = 3)	Main function	*p*
**1. *Serum albumin***	P02768.2	69	79	127.0 ± 48.9	167.8 ± 23.4	140.1 ± 23.5	197.1 ± 16.1	Transport	0.06
**2. *Transferrin***	P02787.3	77	34	17.7 ± 5.5 ab	23.8 ± 1.7 b	15.2 ± 2.7 a	26.6 ± 6.7 ab	Transport	**0.02**
**3. *Apoliprotein A-I***	P02647.1	31	36	9.11 ± 9.5	18.3 ± 9.8	13.7 ± 3.5	23.5 ± 3.6	Transport	0.14
**4. *Haptoglobin***	P00738.1	45	14	14.0 ± 1.4	15.1 ± 12.4	24.9 ± 6.3	12.5 ± 5.1	Immunomodulatory	0.21
**5. *Ig kappa chain C region***	P01834.2	12	…	9.5 ± 4.5	16.2 ± 1.1	18.9 ± 9.1	18.0 ± 1.6	Immunomodulatory	0.19
**6. *Ig gamma-1 chain C region***	P01857.1	36	. . .	9.6 ± 4.8	11.5 ± 2.0	12.9 ± 5.6	8.2 ± 1.58	Immunomodulatory	0.47
**7. *Ig lambda-2 chain C region***	P0DOY2	11	75	8.0 ± 5.1	9.6 ± 4.4	13.5 ± 6.6	9.7 ± 3.6	Immunomodulatory	0.54
**8. *Alpha-2-macroglobulin***	P01023.3	163	05	5.4 ± 3.6	15.9 ± 4.1	8.3 ± 5.9	9.3 ± 4.4	Activate/regulate the complement system	0.07
**9. *Ig alpha-1 chain C region***	P01876.2	38	29	7.2 ± 2.1	8.5 ± 2.3	11.2 ± 3.02	10.1 ± 2.0	Immunomodulatory	0.22
**10. *Alpha-1-antitrypsin***	P01009.3	47	08	3.3 ± 3.1	5.2 ± 1.6	9.0 ± 2.2	5.1 ± 3.6	Activate the coagulation/protease-inhibition pathway	0.08
**11. *Hemopexin***	P02790.2	52	19	3.1 ± 4.0	5.4 ± 2.9	5.5 ± 2.2	8.6 ± 3.0	Transport	0.23
**12. *Ig gamma-2 chain C region***	P01859.2	36	29	1.4± 1.2 b	2.2 ± 1.0 ab	4.5 ± 1.3 ab	4.78 ± 2.5 a	Immunomodulatory	**0.04**
**13. *Alpha-1-acid-glycoprotein***	P02763.1	24	19	2.6 ± 3.0	3.3 ± 3.2	4.9 ± 2.1	3.6 ± 1.5	Transport	0.71
**14. *Complement C3***	P01024.2	187	05	1.1 ± 1.1	4.5 ± 4.0	2.0 ± 1.2	4.7 ± 3.7	Immunomodulatory	0.34
**15. *Apolipoprotein A-II***	P02652.1	11	58	0.4 ± 0.5 b	2.4 ± 2.6 ab	0.8 ± 0.5 ab	4.8 ± 2.1 a	Transport/metabolize lipids	**0.04**
**16. *Ig gamma-3 chain C region***	P01860.2	41	. . .	0.8 ± 1.2	2.0 ± 1.1	0.8 ± 0.6	1.7 ± 1.0	Immunomodulatory	0.34
**17. *Ig gamma-4 chain C region***	P01861.1	36	23	2.8 ± 5.0	1.3 ± 1.6	3.7 ± 5.1	0.8 ± 1.5	Immunomodulatory	0.73
**18. *Vitamin D-Binding Protein***	P02774.1	53	05	0.2 ± 0.3	1.0 ± 1.5	0.7 ± 0.6	1.6 ± 0.5	Immunomodulatory	0.39
**19. *Ceruloplasmin***	P00450.1	122	01	0.0 ± 0.0	1.1 ± 0.6	± 1.1	0.1 ± 0.1	Transport	0.08
**20. *Complement C4-A***	P0C0L4.2	193	01	0.2 ± 0.3	0.3 ± 0.4	0.5 ± 0.9	1.0 ± 0.9	Immunomodulatory	0.46
**21. *Alpha-1-antichymotrypsin***	P01011.2	48	02	0.2 ± 0.3	0.5 ± 1.1	0.6 ± 0.2	0.0 ± 0.0	protease-inhibition pathway/ metabolize lipids	0.56
**22. *Kininogen***	P01042.2	72	02	0.1 ± 0.1	0.5 ± 0.6	0.1 ± 0.1	0.2 ± 0.3	protease-inhibition pathway	0.60
**23. *Ig alpha-2 chain C region***	P01877.3	37	08	0.0 ± 0.0	0.1 ± 0.3	0.0 ± 0.0	0.0 ± 0.0	Immunomodulatory	0.52
**24. *Beta-globin***	P68871.2	16	09	0.3 ± 0.5	2.1 ± 2.0	0.5 ± 1.0	0.3 ± 0.3	Transport	0.22
**25. *Ig kappa chain V-III***	P04433.1	13	08	0.2 ± 0.3	0.8 ± 0.5	0.7 ± 1.5	0.3 ± 0.5	Immunomodulatory	0.78
**26. *Beta-2-glycoprotein 1***	P02749.3	38	15	0.1 ± 0.1	0.6 ± 0.4	0.3 ± 0.6	0.8 ± 0.1	Extracellular matrix	0.22
**27. *Ig heavy chain V-III TIL***	P01764.2	12	26	0.3 ± 0.5	0.0 ± 0.0	1.4 ± 2.2	0.1 ± 0.1	Immunomodulatory	0.41
**28. *Complement factor B***	P00751.2	86	01	0.0 ± 0.0	0.5 ± 0.6	0.4 ± 0.4	0.5 ± 0.5	Immunomodulatory	0.49
**29. *Alpha-globin***	P69905.2	15	11	0.0 ± 0.0	0.5 ± 0.6	0.1 ± 0.1	0.0 ± 0.0	Transport	0.24

Small letters compare values in the same row. The same letter indicates means that do not differ from each other, while different letters indicate statistically significant differences (p≤0.05); AF—acute / subacute form; CF- chronic form and n- number of participants.

The only difference observed in transferrin was a higher level in patients with PCM caused by *P*. *lutzii* than those caused by *P*. *brasiliensis*, without relapse (Table 3).

However, the gamma-2 chain C region and apolipoprotein A-II Ig proteins showed reduced expression in the group of relapsed patients with PCM caused by *P*. *brasiliensis*, in relation to the control group (Table 3).

**Fig 3:**
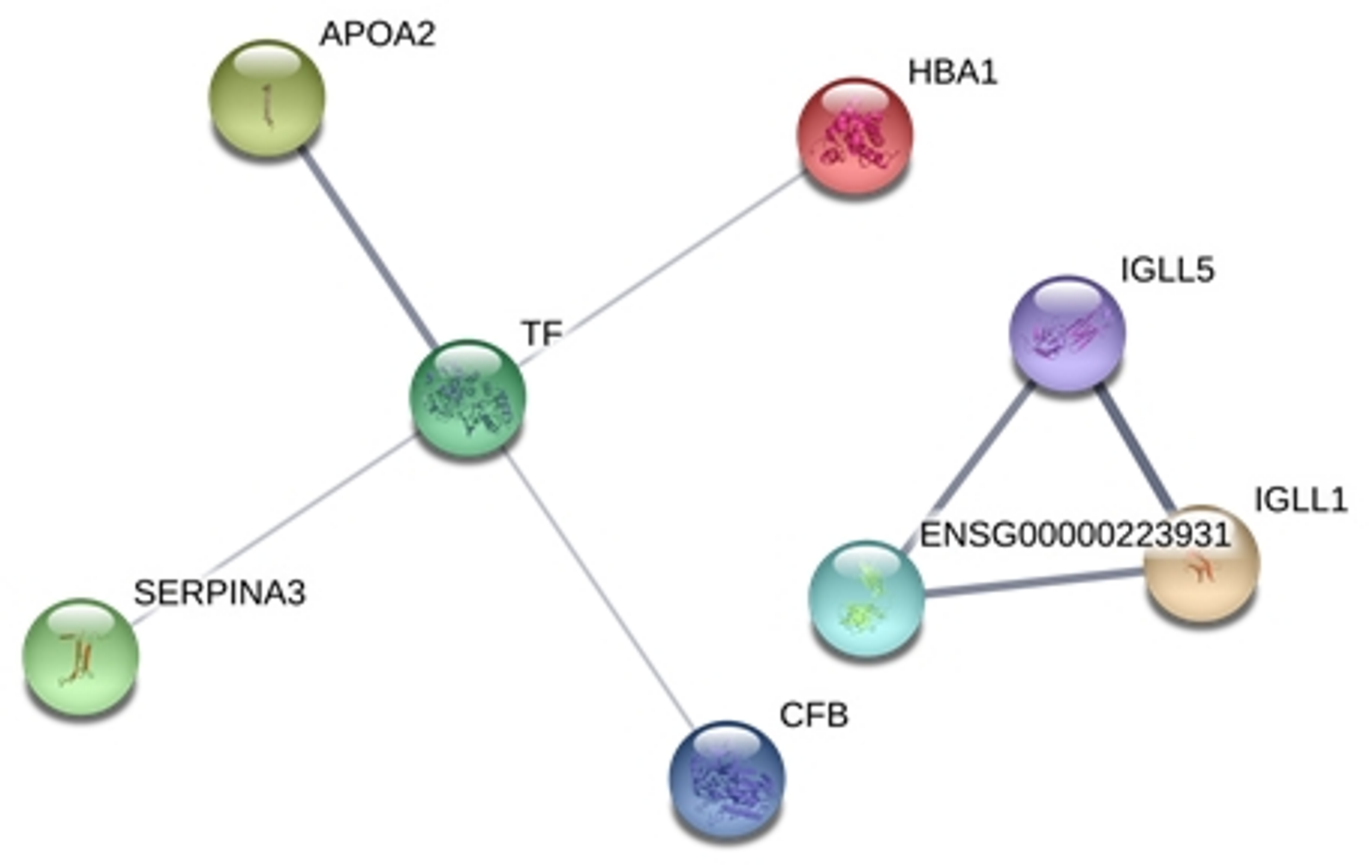
Interaction network between the 8 proteins differential expressed in infections of *P*. *brasiliensis* and *P*. *lutzii* before treatment. The stronger associations are represented by thicker lines. The protein network was analyzed using STRING software. TF: transferrin, APOA2: Apoliprotein A-II, SERPINA3: alpha-1-antichytrypsin, CFB: complement factor B and HBA1: alpha-globin, IGLL1: Ig alpha-2-chain C region, IGLL5: Ig gamma-2-chain C region and ENS000223931: Ig heavy chain V-III TIL.

In this STRING analysis (Fig 3), a strong interaction among the Ig alpha-2-chain C region (IGLL1), the Ig gamma-2-chain C region (IGLL5), and the Ig heavy-chain VIII TIL (ENS000223931) was observed. In addition, interactions among transferrin (TF), apoliprotein A-II (APOA2), alpha-1-antichytrypsin (SERPINA3), CFB, and alpha-globin (HBA1) proteins were also observed.

## Discussion

The proteomic methodology has been used for the identification of many agents of infectious diseases, such as bacteria [17,18], protozoa [19,20] and fungi [21–24]. Some proteomic studies have been performed to identify *Paracoccidioides* spp. isolates regarding its species [7, 13, 25,26]. All these studies focused proteins from the etiological agents, and none of them analyzed protein froms serum samples of the patients.

Some studies have used two-dimensional electrophoresis and mass spectrometry to isolate and identify proteins to be useful in the diagnosis and monitoring of treatment in *Candida albincans* [21,22], *Cryptococcus gatti* [24] and *Aspergillus fumigatus* [23].

PCM is a systemic granulomatous disease that, in spite of an appropriate treatment, can present late relapse due to the persistence of latent fungi. There are two problems to be considered: 1) What would be the best marker to differentiate the proteins caused by *P*. *brasiliensis* from those produced by *P*. *lutzii*? 2) At admission, is it possible to detect the biomarker for a late relapse?

Human serum contains a large number of proteins, classified in relationship to their functions as transporters, immunomodulators, activators, and regulators of the complement system; activators of the coagulation pathway; and as protease inhibitors and lipid metabolism and matrix proteins. Several proteins perform more than one of these functions. These proteins, as well as tissue molecules, can be used in the diagnosis and monitoring of therapy [27]. Although 1,175 proteins have been described in human plasma [28], only 10 of them constitute 95% of the total protein content [29, 30]: albumin (54%); IgG (alpha-1-antitrypsin; 3.8%); alpha-2-macroglobulin (3.6%), IgA (3.5%); transferrin (3.3%); haptoglobin (3%); apolipoprotein A-1 (3%);IgM (2%); and alpha-1 acid glycoprotein (1.3%).

The analysis of patient serum samples drawn at admission revealed the presence of the alpha-1-antichymotrypsin protein (SERPINA 3) in patients with active PCM, with no quantitative difference among those caused by *P*. *brasiliensis*, either with or without subsequent relapse, and those by *P*. *lutzii*. Since alpha-1-antichymotrypsin (SERPINA 3), a protease inhibitor protein that acts on lipid metabolism, is present during inflammatory processes, including those of infectious etiology, these findings corroborate those of the literature [31].

The complement factor B (CFB) is part of the complement system, which is composed of plasma membrane proteins and soluble proteins in blood; these proteins participate in the innate and acquired defenses by opsonizing pathogens and inducing an appropriate series of inflammatory responses. In PCM patients, the contact between the yeast cells and the phagocytes is facilitated by the activation of the alternative pathway of the complement system, which leads to its opsonization [32]. In addition, the fungus itself, including the 43 kDa glycoprotein (gp43), which is considered the main antigen secreted by *P*. *brasiliensis*, can promote the initial adhesion and internalization of the yeast by phagocytic cells [33]. The results revealed that CFB was not identified in the serum of patients who had relapsed, suggesting that the absence of this marker on admission might be predictive of relapse.

Alpha globin (HBA1), a component of the hemoglobin [34], was not identified at admission in patients who later relapsed, such as the CFB. However, HBA1 differs from CBF insofar as it was not identified in the control group.

The Ig alpha-2 chain C region (IGLL1) is an immunomodulatory protein that constitutes the major class of immunoglobulins present in body secretions. It acts against local infection and it also blocks the access of foreign antigens to the general immune system [35]. The identification of this protein only in serum from patients infected with *P*. *lutzii* suggests that IGLL1 could be a biomarker of this etiology. It is worth mentioning that this protein was not identified in control sera.

The heavy-chain Ig V-III TIL (ENS000223931), which participates in antigen recognition [36], was not identified in the serum of patients infected by *P*. *lutzii*, but it was present in those with PCM caused by *P*. *brasiliensis* and in the control group. Thus, the absence of this antigen suggest the presence of PCM caused by *P*. *lutzii*.

Transferrin (TF) is a glycoprotein constituted by a single polypeptide chain and its main function is to transport iron through plasma [37]. Serum TF levels were lower in samples from PCM patients than from those in the control group. Since the fungi of the genus *Paracoccidioides* consume iron [10, 38, 39], our findings confirm those of previous works; however, no differences in TF serum levels were observed among PCM patients from the three groups.

The Ig gamma-2 chain C region (IGLL5) revealed, at admission, a TF-like behavior, whereby IGLL5 presented lower values than those observed in healthy controls. However, different from what was observed for TF, IGLL5 was present in lower levels in relapsed *P*. *brasiliensis* patients than in the unrelapsed ones. It also appeared in lower levels among those infected by *P*. *lutzii*. These findings suggest that patients with very low TF serum levels may relapse.

The strong interaction observed among the Ig alpha-2-chain C region (IGLL1), the Ig gamma-2-chain C region (IGLL5), and the Ig heavy-chain VIII TIL (ENS000223931) was expected because they are proteins of the immune system, active as a defensive barrier against infectious conditions.

The proteomic assay was used to evaluate more than 20 proteins in the same analysis, aiming to use a proteomic approach to identify proteins that could differentiate infections caused by *P*. *brasiliensis* from those produced by *P*. *lutzii*, as well as to identify a biomarker that could predict patients prone to relapse. It is well known that this methodology could not be standardized in the clinical laboratories. Thus, the next step of this research is the evaluation of the selected proteins using commercial kits. A higher number of patients will be evaluated, and the selections of serum samples are ongoing.

This is the first time the proteomic methodology was performed in serum samples to identify biomarkers for infectious diseases. These findings, evaluated at patient’s admission, indicate that the presence of IGLL1 and the absence of heavy-chain V-III TIL Ig (ESP000223931) are indicators of *P*. *lutzii* infection. Moreover, the absence of CFB at admission may be a predictor of relapse.

## Supporting information

S1 Table **(A) Quality control of proteomic data by protein identification probability, presented as average in patients with paracoccidioidomycosis, evaluated before treatment—G1, G2, G3 patients groups and healthy individuals—G4 group.** AF—acute / subacute form; CF- chronic form; n- number of participants; … sequence not in database. (B) **Quality control of proteomic data by protein identification probability, presented as average in patients with paracoccidioidomycosis, evaluated before treatment—G1, G2, G3 patients groups and healthy individuals—G4 group.** AF—acute / subacute form; CF- chronic form; n- number of participants; … sequence not in database. (C) **Quality control of proteomic data by protein identification probability, presented as average in patients with paracoccidioidomycosis, evaluated before treatment—G1, G2, G3 patients groups and healthy individuals—G4 group.** AF—acute / subacute form; CF- chronic form; n- number of participants; … sequence not in database.
(PDF)
