# Supplementary material for: Serological proteomic biomarkers to identify Paracoccidioides species and risk of relapse
Source: PLoS One. 2018 Aug 29;13(8):e0202804. doi: 10.1371/journal.pone.0202804 (PMC6114792; doi:10.1371/journal.pone.0202804)
Supplement: S1 Table — (A) Quality control of proteomic data by protein identification probability, presented as average in patients with paracoccidioidomycosis, evaluated before treatment—G1, G2, G3 patients groups and healthy individuals—G4 group. AF—acute / subacute form; CF- chronic form; n- number of participants; … sequence not in database. (B) Quality control of proteomic data by protein identification probability, presented as average in patients with paracoccidioidomycosis, evaluated before treatment—G1, G2, G3 patients groups and healthy individuals—G4 group. AF—acute / subacute form; CF- chronic form; n- number of participants; … sequence not in database. (C) Quality control of proteomic data by protein identification probability, presented as average in patients with paracoccidioidomycosis, evaluated before treatment—G1, G2, G3 patients groups and healthy individuals—G4 group. AF—acute / subacute form; CF- chronic form; n- number of participants; … sequence not in database. (PDF) [file pone.0202804.s001.pdf]

Table S1A. Quality control of proteomic data by protein identification probability, presented as average in patients with paracoccidioidomycosis, evaluated before treatment (at admission) - G1, G2, G3 patients groups and healthy individuals - G4 group.

Protein	Access code	Molecular mass (kDa)	Coverage rate (%)	[G1] *P. brasiliensis* with relapse AF (n=1) / CF (n=2) (%)	[G2] Patients with *P. lutzii* CF (n=4) (%)	[G3] *P. brasiliensis* without relapse AF (n=2) / CF (n=2) (%)	[G4] Control group (n=3) (%)	Main function
**1.** *Serum albumin*	P02768.2	69	79	100	100	100	100	Transport
**2.** *Transferrin*	P02787.3	77	34	100	100	100	100	Transport
**3.** *Apoliprotein A-I*	P02647.1	31	36	67	100	100	100	Transport
**4.** *Haptoglobin*	P00738.1	45	14	100	100	100	100	Immunomodulatory
**5.** *Ig kappa chain C region*	P01834.2	12	…	100	100	100	100	Immunomodulatory
**6.** *Ig gamma-1 chain C region*	P01857.1	36	...	100	100	100	100	Immunomodulatory
**7.** *Ig lambda-2 chain C region*	P0DOY2	11	75	100	100	100	100	Immunomodulatory
**8.** *Alpha-2-macroglobulin*	P01023.3	163	05	75	100	100	100	Activate/regulate the complement system
**9.** *Ig alpha-1 chain C region*	P01876.2	38	29	100	100	100	100	Immunomodulatory
**10.** *Alpha-1-antitrypsin*	P01009.3	47	08	66	92	100	100	Activate the coagulation/protease-inhibition pathway
**11.** *Hemopexin*	P02790.2	52	19	56	100	94	100	Transport
**12.** *Ig gamma-2 chain C region*	P01859.2	36	29	92	100	100	100	Immunomodulatory

AF - acute / subacute form; CF- chronic form; n- number of participants; … sequence not in database.

Table S1B. Quality control of proteomic data by protein identification probability, presented as average in patients with paracoccidioidomycosis, evaluated before treatment (at admission) - G1, G2, G3 patients groups and healthy individuals - G4 group.

Protein	Access code	Molecular mass (kDa)	Coverage rate (%)	[G1] *P. brasiliensis* with relapse AF (n=1) / CF (n=2) (%)	[G2] Patients with *P. lutzii* CF (n=4) (%)	[G3] *P. brasiliensis* without relapse AF (n=2) / CF (n=2) (%)	[G4] Control group (n=3) (%)	Main function
**13.** *Alpha-1-acid-glycoprotein*	P02763.1	24	19	66	74	100	99	Transport
**14.** *Complement C3*	P01024.2	187	05	25	78	68	89	Immunomodulatory
**15.** *Apolipoprotein A-II*	P02652.1	11	58	43	66	59	100	Transport/metabolize lipids
**16.** *Ig gamma-3 chain C region*	P01860.2	41	...	92	99	96	98	Immunomodulatory
17. Ig gamma-4 chain C region	P01861.1	36	23	84	85	92	89	Immunomodulatory
**18.** *Vitamin D-Binding Protein*	P02774.1	53	05	11	32	32	60	Immunomodulatory
**19.** *Ceruloplasmin*	P00450.1	122	01	0	55	57	11	Transport
**20.** *Complement C4-A*	P0C0L4.2	193	01	4	24	29	44	Immunomodulatory
**21.** *Alpha-1-antichymotrypsin*	P01011.2	48	02	11	25	42	0	protease-inhibition pathway/ metabolize lipids
**22.** *Kininogen*	P01042.2	72	02	11	34	11	11	protease-inhibition pathway

AF - acute / subacute form; CF- chronic form; n- number of participants; … sequence not in database.

Table S1C. Quality control of proteomic data by protein identification probability, presented as average in patients with paracoccidioidomycosis, evaluated before treatment (at admission) - G1, G2, G3 patients groups and healthy individuals - G4 group.

**Protein**	**Access code**	**Molecular mass (kDa)**	**Coverage rate (%)**	**[G1] *P. brasiliensis* with relapse AF (n=1) / CF (n=2) (%)**	**[G2] Patients with *P. lutzii* FC (n=4) (%)**	**[G3] *P. brasiliensis* without relapse FA (n=2) / FC (n=2) (%)**	**[G4] Control group (n=3) (%)**	**Main function**
**23.** *Ig alpha-2 chain C region*	P01877.3	37	08	0	0	0	0	Immunomodulatory
**24.** *Beta-globin*	P68871.2	16	09	26	66	24	31	Transport
**25.** *Ig kappa chain V-III*	P04433.1	13	08	11	56	24	21	Immunomodulatory
**26.** *Beta-2-glycoprotein 1*	P02749.3	38	15	11	39	16	75	Extracellular matrix
**27.** *Ig heavy chain V-III TIL*	P01764.2	12	26	22	0	33	11	Immunomodulatory
**28.** *Complement factor B*	P00751.2	86	01	0	29	32	32	Immunomodulatory
**29.** *Alpha-globin*	P69905.2	15	11	0	25	2	0	Transport

AF - acute / subacute form; CF- chronic form; n- number of participants; … sequence not in database.

DATABASE SEARCHING-- Tandem mass spectra were extracted by [unknown] version [unknown]. Charge state deconvolution and deisotoping were not performed. All MS/MS samples were analyzed using Mascot (Matrix Science, London, UK; version 2.3.02). Mascot was set up to search the Uniprot_Human_canonical_isoforms_release_17032016 database (unknown version, 92180 entries) assuming the digestion enzyme trypsin. Mascot was searched with a fragment ion mass tolerance of 0,100 Da and a parent ion tolerance of 0,100 Da. Carbamidomethyl of cysteine was specified in Mascot as a fixed modification. Oxidation of methionine was specified in Mascot as a variable modification.

CRITERIA FOR PROTEIN IDENTIFICATION-- Scaffold (version Scaffold_4.7.5, Proteome Software Inc., Portland, OR) was used to validate MS/MS based peptide and protein identifications. Peptide identifications were accepted if they could be established at greater than 24,0% probability to achieve an FDR less than 0,1% by the Scaffold Local FDR algorithm. Protein identifications were accepted if they could be established at greater than 99,0% probability to achieve an FDR less than 1,0% and contained at least 2 identified peptides. Protein probabilities were assigned by the Protein Prophet algorithm (Nesvizhskii, Al et al Anal. Chem. 2003;75(17):4646-58). Proteins that contained similar peptides and could not be differentiated based on MS/MS analysis alone were grouped to satisfy the principles of parsimony. Proteins sharing significant peptide evidence were grouped into clusters.
